# Sources of variation in developmental language disorders: evidence from eye-tracking studies of sentence production

**DOI:** 10.1098/rstb.2012.0393

**Published:** 2014-01-19

**Authors:** Courtenay Frazier Norbury

**Affiliations:** Department of Psychology, Royal Holloway, University of London, Egham, Surrey TW20 0EX, UK

**Keywords:** language impairment, autism, eye-movements, language production, visual world

## Abstract

Skilled sentence production involves distinct stages of message conceptualization (deciding what to talk about) and message formulation (deciding how to talk about it). Eye-movement paradigms provide a mechanism for observing how speakers accomplish these aspects of production in real time. These methods have recently been applied to children with autism spectrum disorder (ASD) and specific language impairment (LI) in an effort to reveal qualitative differences between groups in sentence production processes. Findings support a multiple-deficit account in which language production is influenced not only by lexical and syntactic constraints, but also by variation in attention control, inhibition and social competence. Thus, children with ASD are especially vulnerable to atypical patterns of visual inspection and verbal utterance. The potential to influence attentional focus and prime appropriate language structures are considered as a mechanism for facilitating language adaptation and learning.

## 1. Introduction

Speaking in sentences is a momentous developmental milestone that marks the beginning of a child's ability to communicate an infinite array of ideas, feelings and experiences, past and present, real or imagined, to other people. Intuitively, this seems an effortless development for most children, yet producing a fluent and meaningful utterance is a complex process. Models of skilled, adult sentence production outline at least three key processes: conceptualization, formulation and articulation [1]. Conceptualization is the stage at which speakers decide on the message to be conveyed. Formulation requires that speakers map that intended message onto word forms (lexicalization) and particular word orders (syntactic planning). Finally, the speaker must plan and make the motor movements necessary to articulate the message. The vast majority of children will begin to master this complex process in the preschool years. For those with neurodevelopmental disorders, however, language production may present lifelong challenges. Until recently though, we have known little about where in the processing chain language breaks down and how identifying points of difficulty may inform potential intervention strategies.

Traditionally, psychologists and linguistics have had to rely on the product of this processing chain to infer the prerequisites needed at each stage for successful production [2]. What skilled speakers say, and importantly the timing of production elements and the errors that speakers make, have provided insights into processing mechanisms and informed computational models of how meaning is translated into the syntax of a particular language [3]. Such models have also made inroads into our understanding of how children become competent syntactic speakers. These models highlight the importance of experience-dependent and error-based learning mechanisms that are shaped by the innate (neural) architecture of the developing system [4]. For most young children, experience is socially mediated; children learn from their interactions with caregivers and very quickly can adapt their language production to their listening audience. Thus, typical children become competent speakers by evaluating their actual or intended outputs against expectations derived from linguistic input [4] and the social conventions shared by interlocutors [5].

What speakers say, however, will only take us so far. Analysing speech output tells us relatively little about the process of conceptualization, for instance, how do people decide what to talk about and how do children learn to create utterances that are relevant to context and to listener need? How is the form of the intended message constrained by individual differences in the developing system, by for instance, limited vocabulary or reduced memory capacity? This question assumes particular relevance when considering language development in atypical populations; similar output may arise from qualitatively different underlying processing mechanisms (cf. [6]). Recent advances in eye-tracking technology may elucidate these processes in new ways, by taking advantage of the fact that eye-movements are temporally linked to verbal output [7], and thus can reveal more about the processes involved in conceptualization, formulation and articulation of utterances, *as they occur*. This paper therefore considers the application of eye-tracking paradigms to investigate language production in two common neurodevelopmental disorders, autism spectrum disorder (ASD) and developmental language impairment (LI). The goal of the research programme is to elucidate any qualitative differences between these two populations in the conceptualization and formulation of sentences. The work builds extensively on adult models of sentence processing, but yields novel insights into the multiple factors that contribute to language development and disorder. The paper begins by considering what is known from previous eye-tracking studies about language production processes in skilled adult speakers and young typically developing speakers. Then, language production in ASD and LI and the potential advantages of using eye-movements to reveal continuities and discontinuities in the language processing of these populations is discussed. Finally, four case studies of eye-movements and language production in children with typical development (TD), ASD and LI are provided, highlighting possible sources of language breakdown and hinting at future intervention strategies.

## 2. Eye-tracking as a window on the ‘process’ of language production

**Figure 1.**
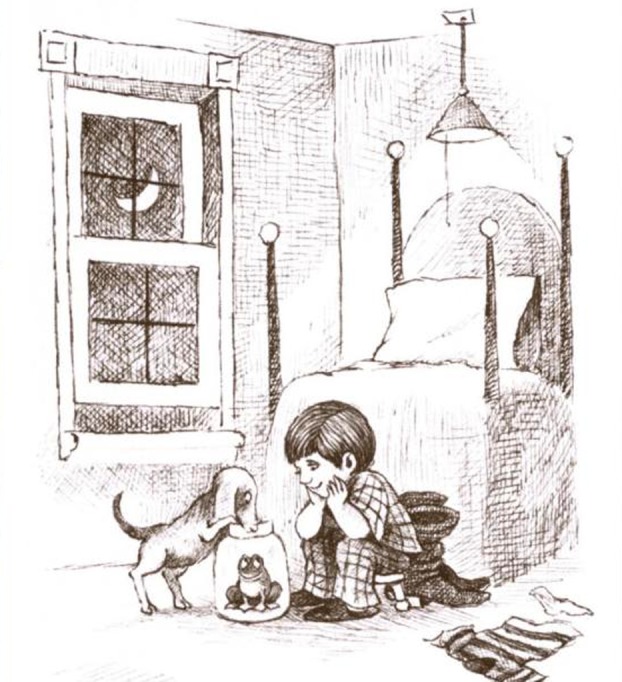
Opening page of the children's picture book, *Frog, where are you?* [8].

Consider the image in figure 1, taken from a well-known children's picture book, *Frog, Where Are You?* [8]. There are numerous ways to describe this scene, among them:

(1) The boy and the dog are looking at the pet frog.
(2) There is a frog in a jar and the boy is smiling at him.
(3) The boy is sitting with his pets.

Extensive research using this book to elicit narratives from children and adults across a range of language communities has shown that the vast majority of speakers begin their story with reference to the boy and one or both of the animals [9]. However, a study of narrative production in children with developmental disorders [10] produced some more unusual opening gambits, the most memorable one from a nine-year-old boy with ASD who started his story with, ‘There is a moon’. While grammatically and factually correct, such a statement violates the social expectation that the story will start with a focus on the main protagonists and may contribute to the higher rates of ‘bizarre’ or ‘irrelevant’ utterances reported to characterize the narrative of individuals with ASD [11].

The question is why did this particular child choose to start his narrative with reference to the moon and what does this tell us about the conceptualization process in developmental disorders? Did he fail to note the social elements of the scene, or did he see them but choose to avoid them owing to social deficits associated with ASD? Was the moon the first thing he saw and was he unable to inhibit mention of it? Did he see both elements of the scene but choose to start with the moon, perhaps as an attempt to provide some setting information about the event? Considering an utterance in isolation does not enable us to determine how speakers decide what to talk about nor how they determine the ultimate form and content of the utterance. Recent developments in eye-movement research using the visual world paradigm [12] provide an opportunity to observe how and when speakers acquire the visual materials needed to generate a message, and how this information relates to the timing, content and form of their utterances [13].

In the visual world paradigm, speakers are presented with a visual image, either of a coherent event or a structured object display and are asked to describe it. Eye-movements and verbal output are recorded, revealing where in the scene speakers were gazing relative to when they mentioned the gazed-upon aspects of the scene in their speech. Griffin and Bock [7] were the first to use this methodology to study sentence generation in skilled adult speakers. They asked speakers to describe simple cartoons depicting transitive events (‘the woman is shooting the man’) and demonstrated a highly consistent pattern of visual scanning, temporally linked to verbal output. Specifically, speakers initially surveyed key aspects of the scene for approximately 300 ms, after which gazes to the agent and patient characters began to diverge. In general, looks to the agent increased and this was invariably the first character to be mentioned in verbal output. As the name for the agent was articulated, gaze shifted to the patient. By contrast, when speakers were asked to simply identify the recipient of the action, looks focused almost exclusively on the patient. Griffin and Bock [7] argued that the pattern of eye-movements supported at least two distinct phases of sentence production: a rapid period of event apprehension, during which speakers comprehend the event, and a longer period of utterance formulation in which the specific lexical items and syntactic forms needed to describe the event are accessed and articulated. Importantly, in the formulation phase, objects are fixated in the order in which they are mentioned, looks at the object occur slightly in advance of articulation, and duration of looking time to objects appears to reflect the ease with which objects may be identified and names retrieved [14,15].

Gleitman *et al*. [16] extended these findings by exploring the extent to which directing attention to aspects of the visual scene influenced how speakers described the scene. Participants were asked to describe similar cartoon events; however, on the majority of trials, a brief, implicit visual cue appeared prior to trial onset in the spatial location occupied by one of the scene protagonists. Cueing successfully directed attention in the majority of trials, such that the first fixation corresponded to the cued location. In addition, cued characters were significantly more likely to be mentioned first in utterances affecting syntactic construction. For example, if the patient was cued, participants were more likely to produce passive constructions (e.g. ‘the boy was chased by the dog’) relative to the uncued condition. The tight link between visual attention and verbal output has been more directly assessed by Coco and Keller [17], who demonstrated that for skilled adult speakers, it is possible to predict which objects in a cluttered visual display will be mentioned in an utterance, and the order in which they will be mentioned, by examining the speaker's visual scan patterns.

Few studies of sentence production in younger, typically developing children have employed eye-movement paradigms. Bunger *et al*. [18] asked 4-year-old children and adults to describe short film clips depicting motion events that included an agent, an instrument and an end path (e.g. ‘the boy skated into the net’). In the initial stages of event apprehension, both children and adults fixated the instrument and path regions of the screen to a similar extent. However, the children were less likely to mention both elements of the scene in their utterances, reporting for example ‘the boy went into the net’ or ‘the boy skated.’ Young, typically developing children did not necessarily mention all aspects of a visual scene, even if those regions of the scene were fixated. Bunger *et al*. [18] argued that this reflects a limited capacity linguistic system, rather than developmental differences in attentional processes.

Eye-movement studies of language production in skilled adults and younger typically developing children highlight the potential applications of this methodology to elucidating qualitative differences in developmental disorders. Exploring eye-movements before the onset of speech can reveal group differences in the initial apprehension of events, and allows us to identify which aspects of a scene attract visual attention and are therefore available to speakers. Examining the order of fixations and the order in which scene items are mentioned as the sentence unfolds can reveal more about formulation processes. These paradigms also allow detailed investigation of timing; longer eye–voice spans (the amount of time between fixating an object and articulating the object name) may reflect greater challenges with lexical access, whereas fixating objects prior to articulation may contribute to more fluent utterances. Furthermore, we can also determine whether individual differences in visual attention affect output, and whether this can be externally modified, by cueing participants to relevant scenes. Thus, eye-movement paradigms provide a unique opportunity to explore why and when language production breaks down, and whether the source(s) of breakdown overlap in apparently distinct clinical populations.

## 3. Heterogeneity in the expressive language skills of children with developmental disorders

Computational models of sentence production have been derived from observations of typical and atypical adult speakers [19], and thus represent the end state of a developmental process. While these models have recently been applied to language acquisition [3], modelling the enormous heterogeneity in development is just beginning [20]. This heterogeneity is perhaps most pronounced in developmental disorders, where language acquisition often follows a protracted, and qualitatively different, trajectory. However, such disorders can elucidate potential constraints on the typically developing system. Two disorders provide a compelling example. ASD affects approximately 1% of the school-aged population [21] and is characterized by pronounced social-communication deficits and a restricted and repetitive repertoire of interests and behaviours [22]. Specific LI, on the other hand, affects approximately 3–7% of school-aged children [23] and is characterized by pronounced impairments in grammatical production which are often, but not always, accompanied by weaknesses in grammatical comprehension and vocabulary development. There is a great deal of controversy concerning the potential behavioural, cognitive and aetiological overlap between these two disorders [24] and an urgent need to establish whether quantitative similarities in performance on linguistic tasks in the two clinical populations is underpinned by qualitative differences in language processing.

The picture is complicated by the extent of language variation within ASD, which is not easily reconciled by theories attempting to explain the locus of LI within this population. LI within ASD is most often attributed to deficits in social interaction and social understanding, which disrupt the input experienced by young children with ASD [25]. For instance, the degree to which toddlers with ASD preferentially attend to child-directed speech predicts both concurrent vocabulary scores and vocabulary growth one year later [26]. The amount and quality of interaction and the child's ability to engage in joint attention behaviours are also significant predictors of language outcome in this population [27]. Impairments in the ability to impute speaker intention have been implicated in word-learning deficits [28], with later implications for the development of syntax [29] and discourse comprehension [30]. Social deficits should also compromise aspects of language production; for instance, reduced understanding of listener needs could contribute to utterances that were underspecified or less relevant to the task at hand [11].

However, any social theory of LI in ASD must explain why a significant proportion of children with ASD acquire age-appropriate (or indeed, exceptional) vocabularies [31] and go on to develop abstract grammar [32,33]. Individual differences in social engagement may explain some of this variance [34], yet numerous studies have demonstrated that children with similar ASD symptom profiles may nevertheless differ dramatically in language outcome [35,36]. This raises the possibility that children with ASD who develop structural language skills within the normal range (‘autism language normal’ or ALN) may rely on non-social cognitive strengths to compensate for fundamental social weaknesses. For example, verbally able individuals with ASD are reported to have enhanced perceptual and/or attentional abilities [37,38], particular facility with phonological processing [39] and enhanced aspects of declarative learning, that support lexical and semantic development [40]; see also [41] for review.

A substantial proportion of children with ASD, however, have structural language attainments that are significantly below chronological or mental age expectations [31,35], while a significant minority fail to develop meaningful phrase speech [42]. While these children may have poorer social abilities, at least early in development, it is also possible that they experience co-occurring deficits in those aspects of perception, attention, phonological processing and learning that protect their ALN peers from more widespread LI. There is debate regarding whether children with ‘autism and language impairment (ALI)’ constitute a distinct neurocognitive phenotype, in which LI is a co-morbid deficit with similar origins to that seen in specific LI [24,43]. For example, at a group-level children with LI and ALI have difficulties with non-sense word repetition [44], tense-marking of verbs [45] and resolving ambiguities during sentence comprehension [46,47], though qualitative differences between groups in error profiles are often evident. These qualitative differences in output raise questions about whether language deficits in ALI arise from the same cognitive constraints thought to characterize LI or whether they arise from different underlying mechanisms [48].

In general, any social difficulties that exist within LI are thought to be a consequence of negotiating the social world with LI, which may disrupt social interactions and peer relationships [49]. Theories of LI have focused on reduced processing capacity or generally slowed processing, in which lexical access and syntactic planning are slow and effortful, resulting in reduced ability to learn rules from the relevant input [50]. Alternatively, the procedural deficit hypothesis [51] posits that impaired development of the neural circuits that support procedural learning prohibits the implicit learning of rule governed behaviour, including grammar [52].

Thus, children with ALN, ALI and LI have patterns of linguistic and social behaviour that overlap in some domains, but differ in others. There are hints that similarities in language performance arise for qualitatively different underlying reasons, but little direct evidence that this is the case. Eye-movements may therefore further elucidate diagnostic distinctions among these clinical populations. Few eye-tracking studies of language processing in ASD exist, and those that do have focused on monitoring of social cues, such as eye gaze and gesture in communicative contexts [53,54]. One study has investigated online language comprehension in children with ASD and peers matched for language ability [46]. Adolescents observed four items in a display (e.g. *hammer, hamster, candle and cannon*) while listening to sentences that were either neutral (‘John chose the … ’) or biased one of the objects (‘John stroked the … ’). In the neutral condition, there is an equal probability of gazing at any of the four objects at the point the verb unfolds. When ‘ham … ’ is uttered, we expect looks to increase to both the *hammer* and *hamster*, yielding a temporary ambiguity until ‘ … ster’ is processed and looks increase to the target *hamster*. By contrast, the biased condition ensures early gaze to target and minimal looks to any of the distracters, including the *hammer*, because the *hamster* is the only strokeable object in the display. Indeed, Brock *et al*. [46] found that all groups demonstrated anticipatory gaze toward the target in the biased condition. However, when the target *hamster* was not visually displayed, participants with LIs were unable to use the biasing context to inhibit looks to the *hammer*, a phonologically similar competitor. In other words, children with LI and ALI increased fixations to the *hammer* when they heard ‘John stroked the hamster’ even though it was not contextually appropriate to do so, whereas ALN and TD peers did not. In this experiment, ALN and TD peers did not differ on any eye-movement variable. However, differences between children with an ALN profile and TD peers have been reported when participants must use experiential knowledge to guide visual search. Loth *et al*. [55] recorded eye-movements as participants gazed at a cluttered visual scene. Prior to the picture display, participants heard a story that described an event that could be relevant to the scene, for example a picture of a sitting room paired with a story about a burglary or a birthday party. During the first fixations to the visual scene, TD individuals were more likely to fixate scene items that were directly related to the story they had heard, whereas peers with ASD gazed more at neutral items that had little bearing on the narrative context. The authors concluded that top-down attentional control mechanisms may be diminished in ASD.

At the time of writing, no eye-tracking studies of language production in these clinical populations exist. However, combining evidence from studies of skilled adult speakers with what we know about the profiles of strength and weakness across clinical groups leads to some novel hypotheses about processing in these groups. Specifically, a core social deficit and differences in attention control are likely to have a more pronounced effect at the conceptual level of event apprehension and message planning, the ‘what’ of sentence production. Thus, at this stage of language production, children with ASD, regardless of language phenotype, are predicted to resemble one another and show significantly different fixation patterns relative to TD and LI peers. However, once a message has been selected, differences between language phenotypes should become more pronounced. To the extent that ALI and LI represent overlapping aetiologies, we might expect similar difficulties with language formulation. One might predict that generalized slowing will negatively impact on accessing known lexical and syntactic information, resulting in longer speech latencies and disruptions to the timing of sentential elements relative to eye-movements.

## 4. Eye-say: investigating language production processes in typical and atypical development

These predictions are currently being tested in a series of experiments that directly compare language production in children with ALI, ALN, LI and TD. Our first experiment considered potential group differences in volitional control of eye-movements [56]. Previous research has provided conflicting evidence of oculomotor deficits within ASD, though increased variation in saccade accuracy has been reported in only those individuals with ASD who also have LI [57]. In our sample, when tasks simply required reflexive orienting to a visual cue, there were no group differences in speed or fixation accuracy. However, when volitional control of eye-movements was required, both the ALI and the LI groups made more errors than ALN or TD peers. For example, in an antisaccade task, in which viewers must inhibit looks to the salient cue and instead fixate the contralateral location, children with ALI and LI looked more at the salient cue. In addition, when asked to maintain fixation on a target, children with LIs were more likely to fixate distracter items. The source of these errors is uncertain; while in our study language may serve to enhance executive control of eye-movements by providing a mechanism for refreshing rules or task goals in working memory, there is a rich literature suggesting that anomalies in eye-movement control are present in infants and toddlers who are at high risk of developing ASD [58], and their unaffected, first-degree relatives [59]. It has been suggested that early disruptions to visual attention processes may disrupt the development of joint attention, with cascading effects on language development [60].

**Figure 2.**
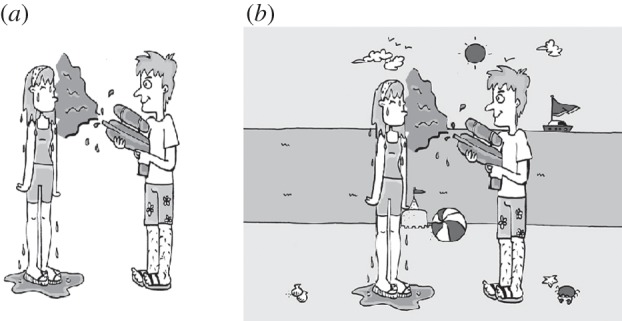
The left image (*a*) depicts a transitive event with minimal objects in the background. The right image (*b*) depicts the same event embedded in a contextually appropriate but visually cluttered scene.

Could variations in visual attention control affect scene scanning and scene description? The majority of eye-tracking studies of language production have presented an event against a plain background, devoid of any extraneous visual context, as illustrated in figure 2*a*. An interesting question, therefore, is whether the same intimate links between visual fixations and verbal output hold when there is more competition for visual attention. Although Coco & Keller [17] presented viewers with cluttered scenes, they provided overt cue words prior to sentence production, which likely guided top-down attention processes. Bunger *et al*. [18] presented cartoon films which included a rich visual context; however, the movement in these films will have increased the salience of the relevant features for description [61].

With regard to ASD, an inability to maintain fixations in the presence of competing stimuli could increase looks to irrelevant objects in the background. Huettig *et al*. [62] suggest that fixating visual objects automatically activates the relevant semantic structures and phonological forms for such objects, thus increasing competition for items to be named in output. Such difficulties should be most evident in unconstrained viewing tasks in which scenes include background information, for example, the image in figure 2*b*. Huettig *et al*. [62] argued that such a relatively unconstrained task may influence visual selection; if there is no *a priori* target to guide scene inspection, irrelevant information may come to the fore. This may be especially detrimental to individuals with ASD, who have ‘enhanced’ perceptual capacity to become aware of items in the background [38], coupled with a reduced preference for socially salient stimuli [58] and difficulties suppressing irrelevant linguistic activations [63]. Together, these propensities may increase both fixations to visually salient items in the background and the likelihood that such items will be mentioned in output. This could help to explain why the child with ASD described figure 1 unusually as ‘There is a moon’.

Table 1 describes four case studies, one for each of the groups of interest. These participants were asked to describe 15 images of transitive events embedded in a contextually appropriate background, as illustrated in figure 2*b*, while their eye-movements were recorded binocularly at a sampling rate of 60 Hz using a Tobii T120 eye tracker. Verbal descriptions were coded as ‘canonical’ or ‘non-canonical’ and the timing of each sentential element was recorded relative to the start of the trial. Canonical utterances were those that described the transitive event and mentioned both the agent and the patient. Utterances that included hesitations, false starts and repetitions were also included in this group. Utterances that contained conjoined noun phrases (‘the boy and the girl are playing on the beach’), passive constructions (‘the girl was soaked by the boy’), or only one character noun phrase (‘someone has a water gun on the beach’) were coded as non-canonical. Timing of sentential elements was then compared to the eye-movement record. Fixations were categorized as occurring prior to speech onset, after speech onset but prior to mention of the sentential subject and after articulation of the subject noun phrase. Fixations were also allocated to one of four scene regions: the agent, the patient, the event core (the area of the image that signalled the event; in figure 2*b* this would be the water gun) and the background (everything else).

**Table 1.** Characteristics of four case studies, including their descriptions of figure 2*b*.

case group	age	total number of canonical utterances (maximum 15)	total number of fixations (15 trials)	percentage fixations to background (%)	number of extraneous items mentioned in output	description of image in figure 2*b*
TD	10.5	14	88	27	0	‘The boy's squirting the girl’.
ALN	8.9	5	287	51	17	‘This boy's splurted this girl with sour milk on the beach’.
LI	13.5	2	166	47	14	‘Someone has a water gun by the beach and the sun is shining out’.
ALI	11.7	12	402	52	22	‘A man with a water pistol, and a … hairy legs, crab, clam shell, squirting this girl and she's not liking it’.

**Figure 3.**
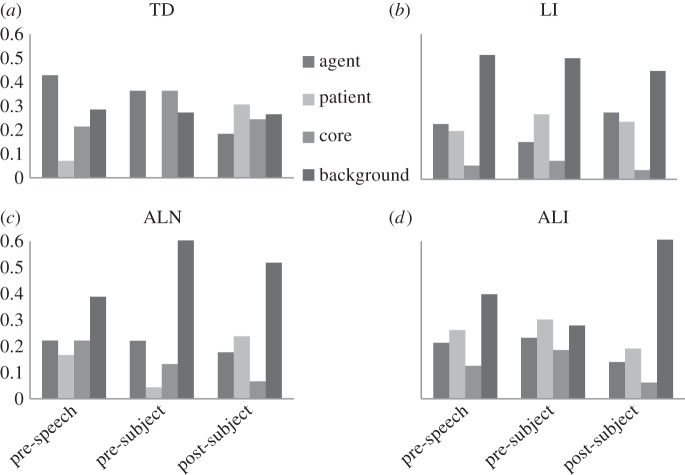
Graph depicting proportion of fixations to scene elements at different points of an utterance for four case studies. Scene elements included the agent (instigator of the action), patient (recipient of the action), event core (key bit of action) and background (all other areas of the screen). Case studies include a child with TD (*a*), LI (*b*), ALN (*c*) and autism plus additional language impairment (ALI: *d*).

Figure 3 illustrates where the four children were looking as the sentence unfolded. The TD participant demonstrates a pattern of eye-movements that is consistent with the skilled adult speakers reported by Brock & Griffin [7]; prior to subject onset there are a greater proportion of fixations to the agent (who then becomes the sentential subject). As the subject is articulated, fixations shift to the patient, the latter mentioned character in verbal output. Thus, to some extent fixations mirror verbal output. However, it is notable that 27% of the TD participant's fixations are to items in the periphery, yet these are never mentioned in output. Thus, typically developing individuals may fixate items in the background, but inhibit mention of these items if they are not deemed relevant to the task at hand.

By contrast, all three children with a clinical diagnosis were more likely to fixate the background, and then mention items peripheral to the central event. This is consistent with recent work demonstrating that once linguistic information is activated, it is difficult for verbal individuals with ASD to inhibit items that are less relevant to the given task [63]. The less typical viewing pattern is mirrored here by an increase in non-canonical utterances. As can be seen from the examples in table 1, these utterances are not necessarily wrong, but do arguably violate our social expectations that speakers provide optimal, relevant and accurate information. The nature of the non-canonical utterances is also revealing. For the child with ALN, 40% of errors were either passive constructions, or cast the patient as the sentential subject. By contrast, 85% and 100% of the non-canonical utterances made by the children with LI and ALI, respectively, involved the omission of a key sentential element, either the verb or the patient. For the child with LI, if an item in the periphery was mentioned, other elements of the sentence were likely to be omitted, yielding utterances that were more like a list of objects rather than a coherent event. This result is reminiscent of Bunger *et al*. [18] and suggests that for these children, a limited linguistic capacity is a constraint on verbal output. What tips the balance such that more peripheral items are mentioned at the expense of core information is an empirical question, but may be influenced by the visual salience of competing stimuli or the ease with which different lexical items may be accessed and articulated.

Neither child with ASD prioritized fixations to the agent or patient at any time point, in stark contrast to the TD child. This may reflect the consistently reported finding of diminished gaze to socially salient stimuli in individuals with ASD (cf. [58]), but may also be influenced by poorer visual attention control. The child with LI shows a similar predominance of fixations to the background but does not have the same profile of social impairment that characterizes the peers with ASD. An interesting question is whether fixating the background affects event apprehension and message conceptualization, or whether these distractions exert a greater influence on message formulation. In truth, it is likely to be both. Clearly, differences in viewing patterns between these ASD participants and the TD child are apparent prior to the onset of speech. However, most fixations occur after speech onset, and as seen in the ALI example, mention of background items appears to be ad hoc, occurring as these items are fixated. It therefore seems unlikely that for these children, the entire message is conceptualized prior to starting the utterance, but rather evolves as they continue to scan the scene.

These examples suggest that children with neurodevelopmental disorders scan complex visual scenes differently to typical peers. Scanning patterns may be influenced by social preferences and variations in attention control. Notably, in this example although the TD child did fixate objects in the background, they were never mentioned in output. Given that fixating items likely activates lexical information about those items, this would suggest that inhibitory control is also an important factor in language production, and one that is also likely to be vulnerable in developmental disorders. If this is the case, guiding children's attention to the relevant aspect of the scene for description may facilitate message formulation. Gleitman *et al*. [16] demonstrated that it was possible to cue visual attention by presenting a brief flash in the relevant spatial location prior to image display. Cueing a particular entity increased the likelihood that this character would become the sentential subject, thus altering preferred syntactic constructions. Specifically, if the patient was successfully cued, the number of passive constructions increased. In our current work with children, spontaneous passives were extremely rare; only 15 out of 1170 utterances were passives. Our laboratory is currently investigating whether directing attention alone would alter syntactic structures for children with developmental disorders.

Computational models of language acquisition [3] suggest that priming and implicit learning are a key mechanism for learning new syntactic forms. Thus, there is potential to direct child's visual attention to relevant aspects of a visual scene, and then provide the child with relevant language input. This should prime more syntactically complex or semantically relevant verbal descriptions of social events. There is evidence that children with specific LIs [64] and children with ASD [65] can be primed to use particular sentence structures, though it is not clear that this extends to syntactic structures that are not already spontaneously used (i.e. passive sentences). In addition, no previous work has explored whether cueing attention and providing targeted language input can increase relevant utterances while at the same time decreasing mention of extraneous items in the periphery. If this were possible, attention cueing and priming in combination could provide a powerful intervention strategy for developing syntactic and narrative skills.

## 5. Summary and conclusion

Adult models of language production have delineated different stages of processing in which potential messages are conceptualized, the lexical and syntactic forms required to encode the message are selected, and the utterance is articulated. Computational models of language acquisition postulate that learning occurs when verbal output is evaluated against linguistic and social expectations and adapted accordingly [4]. Adult studies of language production using eye-movement paradigms have demonstrated the ability to observe the processes that underlie message conceptualization and formulation [7]; application of these methods to developmental disorders can elucidate qualitative differences between clinical populations at the different stages of language production. The example case studies described here suggest that any model of typical and atypical language production will need to take non-linguistic factors, such as attention control, inhibition and social motivation, into account. Such deficits may lead a child to perceive the world differently, resulting in qualitative differences in language input. Children with multiple deficits may therefore find it difficult to learn what is socially relevant, what semantic information is most important to convey and what syntactic forms convey meaning in an unambiguous way. While it may be possible to direct children's attention to relevant features for description, children may not alter their descriptions spontaneously. However, combining attention cueing with sentence priming may provide a powerful mechanism for facilitating language development in clinical populations.
